# Integrity Monitoring of Multimodal Perception System for Vehicle Localization

**DOI:** 10.3390/s20164654

**Published:** 2020-08-18

**Authors:** Arjun Balakrishnan, Sergio Rodriguez Florez, Roger Reynaud

**Affiliations:** CNRS, ENS Paris-Saclay, Université Paris-Saclay, 91190 Gif-sur-Yvette, France; arjun.balakrishnan@universite-paris-saclay.fr (A.B.); roger.reynaud@universite-paris-saclay.fr (R.R.)

**Keywords:** multimodal data source, integrity assessment, intelligent vehicles, localization, Protection Level markers

## Abstract

Autonomous driving systems tightly rely on the quality of the data from sensors for tasks such as localization and navigation. In this work, we present an integrity monitoring framework that can assess the quality of multimodal data from exteroceptive sensors. The proposed multisource coherence-based integrity assessment framework is capable of handling highway as well as complex semi-urban and urban scenarios. To achieve such generalization and scalability, we employ a semantic-grid data representation, which can efficiently represent the surroundings of the vehicle. The proposed method is used to evaluate the integrity of sources in several scenarios, and the integrity markers generated are used for identifying and quantifying unreliable data. A particular focus is given to real-world complex scenarios obtained from publicly available datasets where integrity localization requirements are of high importance. Those scenarios are examined to evaluate the performance of the framework and to provide proof-of-concept. We also establish the importance of the proposed integrity assessment framework in context-based localization applications for autonomous vehicles. The proposed method applies the integrity assessment concepts in the field of aviation to ground vehicles and provides the Protection Level markers (Horizontal, Lateral, Longitudinal) for perception systems used for vehicle localization.

## 1. Introduction

The second half of the last decade has seen a significant emergence of commercially available vehicles with autonomous driving capabilities. We can confidently say that the status of autonomy in vehicles is well into the realm of Society of Automotive Engineers (SAE) level 2 [1]. While the researchers and industries are rapidly moving towards SAE level 3 systems that can dramatically improve driving safety and efficiency, monitoring the integrity of sources and process used in such systems can often pose challenges [2]. In [3], the classical integrity concepts used in aviation are transposed to integrity requirements for ground vehicle localization. Using road-safety-related statistics and geometry of roads and vehicles, [3] derived bounds for localization error in both highway and urban scenarios. They further distributed the derived total integrity risk to allocate integrity levels to every subsystem present in autonomous vehicles. In this work, we focus on the integrity assessment of perception data sources such as vision, LiDAR, map, etc. Most advances in this area explicitly address the task of integrity monitoring of data sources by introducing redundancy in sensors [4,5], using sensors with advanced features [2,6], monitoring repetitive journeys [7], or assuming one source (often high-quality digital maps) as reliable ground truth [8,9]. While adding data redundancy (often different GPS receivers for map-matching and sensor fusion [5]) can monitor the integrity of processes, the integrity of data sources has to be largely assumed. Only a small number of works like [10] and [7] consider digital maps as a source with probabilities of error. However, to achieve context-aware autonomous navigation, perception sensors such as camera and LiDAR are used along with digital maps, GPS, and proprioceptive sensors. In [11], facades of buildings at intersections are detected using vision and are fused with building footprints extracted from the digital map to provide better localization. They further extended their work in [12] to achieve localization at intersections using road structures instead of building facades and map data. A map-matching-based localization involving lane detection from vision is used in [13] and [14]. Similar strategies are employed combining digital maps with features detected from LiDAR data such as curb detection [15], intersection structure detection [16], lane detection [17], etc. However, to the best of our knowledge, integrity monitoring of data from such spatial perception sensors used in the aforementioned works is largely overlooked. Considering the multimodality of data provided by this wide variety of sensors, finding a common framework to evaluate integrity is a challenging yet crucial task. In [18], we made an effort to address this task using a cross-consistency-based integrity monitoring framework for highway scenarios. In this paper, we address limitations of [18] and improve the framework to apply it to complex semi-urban and urban scenarios in a generalized way, thus providing context awareness to a multimodal vehicle localization system.

## 2. Problem Statement

Semi-urban and urban environments often contain a multitude of intersections, roundabouts, road-splits, and merges compared to highway scenarios. As discussed in Section 1, multimodal data from different sources are used to achieve accurate localization in such scenarios. Developing upon the framework presented in [18], finding a generalized common model for the representation of data from all sources is the primary objective of this work. Even though works like [12] and [17] propose geometrical models for several types of intersections, they are limited to a single perception data source and digital maps. They also require prior classification of intersections to reliably fit the predefined models to the data. On the other hand, sensors used in intelligent vehicles have considerably different behavior and output in such scenarios. Hence, the rest of this section is focused on how data from different sources are used in complex scenarios. We also examine the possible errors associated with these use cases and discuss the applicability issues of a simple common geometrical model (e.g., the polynomial model in [18]) in these situations.

Traditionally, vision data is used to detect ego lane markings and/or lanes parallel to the ego lane using a curvature-based model. In urban scenarios, such lane detection models fail due to different types of lane markings (e.g., stop lines, road separation markings, etc.), orientation (e.g., lane markings from other road sections in the junctions) and complex curvatures (e.g., splitting and merging lane markings). Another approach using visual data is to detect the drivable road region in front of vehicle. But due to the unforeseeable shapes of possible road segment detections, modeling of such output with a geometrical model is difficult. Intersections with multilane branch roads can have a large common region at the center, which can limit the observability of other road branches through visual inputs.

It is reasonable to assume that vehicles travel slowly and stop more often in semi-urban and urban scenarios than highways. GPS receivers are proven to have poor performance in slow-moving vehicles [19]. Combined with the fact that the presence of buildings and other obstructions can cause multi-path effects or even outages of signals [2], GPS receivers experience classical localization problems in urban environments.

**Figure 1:**
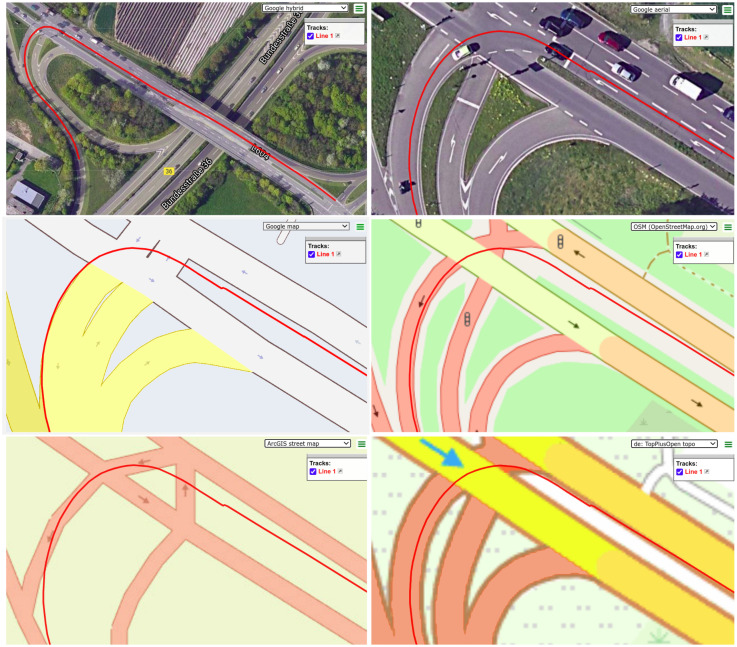
Integrity issues in map sources. Top-left: An example of a GPS track (in red) from the KITTI dataset projected on a satellite map from Google. Top-right: Zoomed aerial view of the track at an intersection. Middle-left: The intersection in a street map from Google. Middle-right: The intersection in a street map from OpenStreetMap. Bottom-left: The intersection in a street map from ArcGIS. Bottom-right: The intersection in a street map from the Federal Agency for Cartography and Geodesy (BKG) of Germany.

With the exception of a few advanced and proprietary Geographic Information Systems (GISs, e.g., Google maps), publicly available GIS sources lack accurate road properties (lane or road widths, locations of lane splits and merges at junctions, etc.) and strongly depend on rule-based rendering to display maps. The discrepancies observed while overlapping the satellite view and rendered map structures from different GISs as shown in Figure 1 are examples of the limitation of this approach. GPS tracking of the vehicle is accurate in satellite view of the junction, which includes a lane change to the leftmost lane of the highway for a left turn and a smooth turn through the left side of link road. However, from the rendered road structure view of all the map sources, the track section corresponding to lane change appears to be wrong as it is outside the boundary of the road structure. It is also worth noticing that none of the GIS represent roads with their actual width, but with rule-based dimensions. It is evident from the same width of two highway sections despite different number of lanes in each of them. Likewise, modeling of junctions is also considerably different in each map source, particularly between Google Maps and OpenStreetMap. Hence, inclusion of map data in localization process is suboptimal in urban and semi-urban scenarios and forces us to consider it as a data source with associated instantaneous integrity rather than ground truth.

While data from vision, GPS, and maps add complexities and impose limitations, LiDAR, on the other hand, can provide useful data in urban and semi-urban environments. It can observe the ego road and other road branches efficiently. By using the reflectivity information available in LiDAR data, we can detect bright surfaces like lane markings and curbs [15]. Though LiDAR poses challenges in the detection and modeling of features as in the case of vision, the accurate 3D information available makes it an important source for representing the structure of a large urban scenario.

The integrity monitoring method in [18] provides a weighting scheme for data sources that infers the cause of inconsistencies observed in the data-fusion method at a given time. For any data source combination that can be represented in a common frame and with a common model in that chosen frame, the cross-consistency analysis proposed in [18] can be applied. However, the discussion presented in this section shows that developing a common model is difficult when different sensor modalities and diverse features are introduced to the system in order to accommodate urban scenarios. To this extent, we could not find any integrity assessment solution in the literature that can handle more than two perception data sources and a wide variety of scenarios.

### Contributions

The paper presents the following contributions based on the problem statement outlined above.

1. Defining a common reference frame and formalizing a common model to represent all data sources in all scenarios.
2. Prototyping an integrity assessment framework using the common model and providing proof of concept.
3. Analyzing the performance of the proposed framework using publicly available datasets and comparison with other state-of-the-art integrity monitoring solutions from the literature.

## 3. Methodology

**Figure 2:**
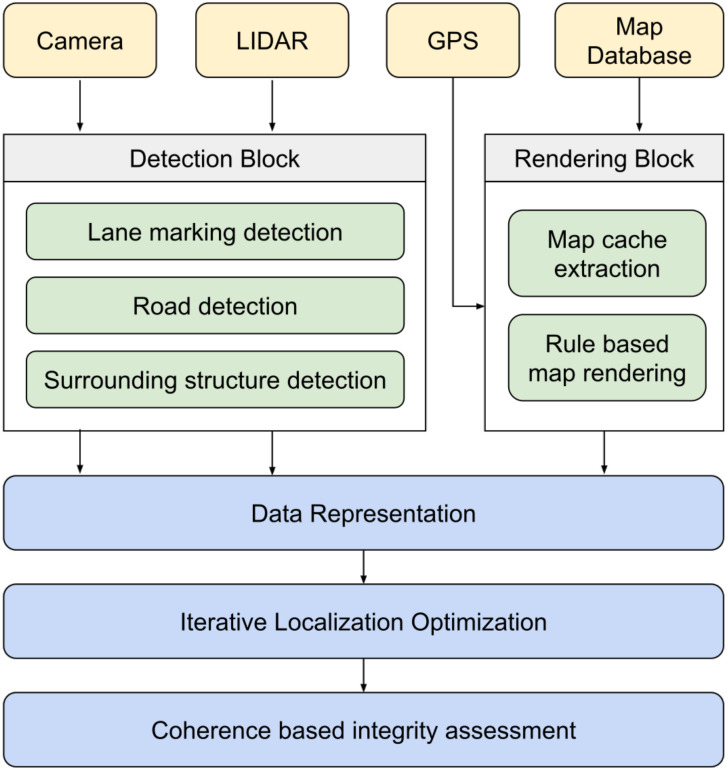
Framework for integrity assessment of multimodal data sources.

The framework proposed for the integrity assessment developed in this work is given in Figure 2. The Detection Block includes sensor-specific routines to detect features that are relevant to different data fusion algorithms described in Section 2. The Rendering Block uses GPS position to extract data from surrounding map regions and applies rule-based rendering to reconstruct the geometrical structure of the area. The obtained information is represented in a common frame using a common model. In this work, the common reference frame is chosen as the ego frame of the vehicle as the transformations between ego frame, camera frame, LiDAR frame, and GPS frame can be determined by calibration procedures [20]. A decision algorithm is used to decide whether the optimization of localization is required in case of unknown transformations between frames of data and the common reference frame (in our case, map frame to ego frame). Once the required optimization is achieved, coherence between data representations is evaluated and integrity is assessed for each source. In this section, we outline the specific techniques and concepts used in the framework presented in Figure 2.

### 3.1. Detection

The purpose of the Detection Block is to extract the same information (features) from each data source. From the literature review, we identify three features that are most commonly used in state-of-the-art localization methods in urban scenarios—lane markings, drivable roads, and the structure of the surroundings of the vehicle. Here, we limit the surrounding structures to grass patches/vegetation and curbs and avoid building facades and other objects due to the complexities of their detection. Indeed, any feature can be used in this process if it is detectable from every data source considered. The methods used to detect these features from each source are explained here.

#### 3.1.1. Vision

To accommodate varieties of lane markings present in different scenarios, all possible markings are detected. Images from cameras are transformed to bird’s-eye view (BEV) using camera calibration. Intensity-based segmentation is used to detect all possible white lane markings. After detection of all the candidate lane markings, blob analysis is used to reject poor detections [14]. Seed-based wavefront segmentation is used to detect dark road regions with asphalt and regions with grass patches. For road segmentation, seeds are selected in front of the vehicle and using propagating waves from these seeds, connected road regions are segmented. Seeds for grass patch detection are selected by color-based keypoint detectors. After these detections, every pixel in the BEV can be classified into lane markings, roads, other surfaces, or unclassified.

#### 3.1.2. LiDAR

A subset of LiDAR data containing points that lie inside a 3D region of interest (ROI) is selected. Points on the road and on the edges of the road are classified using 3D gradients. The ROI is divided into smaller patches in the XY plane, and the points belonging to each patch are examined for their Z values. This helps to differentiate between road segments, curbs, dividers, vegetation, etc. using the technique presented in [21]. Points with high reflectivity are also selected as they correspond to the bright surfaces such as lane markings and railings. These are further classified into reliable lane marking detections by combining their position with road regions. As a result of these detection steps, every point in the ROI is classified with lane markings, roads, other surfaces, or unclassified.

### 3.2. Map Handling

OpenStreetMap (OSM) is used in this work as a GIS source. OSM provides nodes corresponding to ways, grass patches, and railings, etc. However, finding relevant geometrical information in a vehicle’s surroundings from maps involves two key components: location and orientation of the vehicle [10]. Using location measurements, all of the relevant map nodes in the ROI are selected and the map data is transformed into the vehicle’s ego-frame using the orientation of the vehicle. The location estimate is provided by the GPS sensor, whereas the orientation estimate is given by the on-board Inertial Measurement Unit (IMU). Once the map nodes are represented in ego frame, a rule-based rendering algorithm is used to create a geometrical sub-map for the ROI. The number of lanes, lane width (when available), location of road boundaries, boundaries of curbs, dividers, and vegetation, etc. are used in the rendering process, producing an enriched geometrical model of the environment from OSM. In works like [13,14], custom-made high-definition maps (HD maps) that contain lane marking information and accurate road structure information are used. Even though the exact location or type of lane markings are unavailable in OSM, assuming continuous lane markings on the left side of the leftmost lane, right side of the rightmost lane, and dashed lane markings for the lanes in the middle, approximate lane level information can be produced. In case of missing lane width information, the standardized road construction guidelines of the country are used to render the map. However, it is evident that errors in GPS positioning or orientation estimation can greatly affect the accuracy of map data extraction and cause uncertainties in map rendering [10], especially for the exact locations of lane markings.

### 3.3. Representation

**Figure 3:**
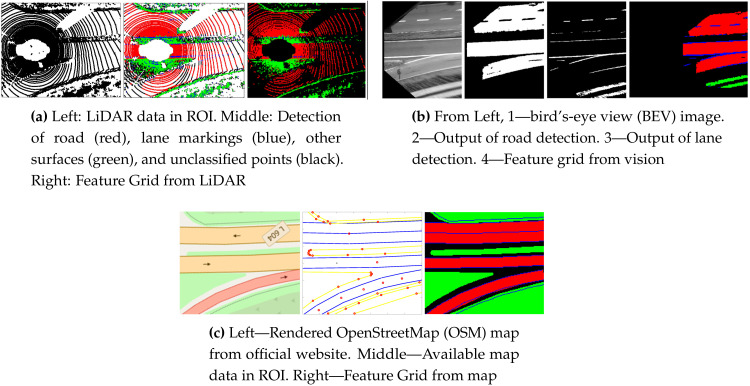
Example of modeling data from different sources using feature grid representation: cells with road labels (red), cells with lane marking labels (blue), cells with other surface labels (green), cells with unclassified labels (black).

To be able to deal with the features and geometries of different types and shapes, a 2D feature grid (FG) is proposed as the model. FG consists of an array of cells where each cell c_i_ represents a 20 × 20 × 100 cm block in the real world. Four feature labels (LBs) are assigned to cells in the FG according to the type of feature: (1) LB_r_—road, (2) LB_l_—lane marking, (3) LB_o_—other surfaces, (4) LB_u_—unclassified/unidentifiable. The blocks corresponding to each of the cells are examined for the information they contain. The type of feature with the highest ratio inside a block is used to assign the respective label to the cell. Each data source produces an FG following this criterion, as shown in Figure 3.

Along with labels, it is important to model the confidence of data provided by each sensor. The accuracy of LiDAR data decreases as the distance from the sensor to the measurement location increases [22]. On the other hand, the Inverse Perspective Mapping (IPM) transformation used to create the bird’s-eye view images from actual images increasingly introduces deformation as the distance from the camera increases due to camera calibration errors. To account for these facts, a confidence function is proposed, drawing inspiration from [18] for all relevant FGs. Using the concept of an Inverse Distance Weighting (IDW) function presented in [23], the weights are computed as

(1) $$w_{ij}=1–{\langle \sqrt{(x_{ij}^{2}+y_{ij}^{2}+h_{s}^{2}}\rangle }_{min-max},$$

where $w_{ij}$ is the weight associated with the cell $C_{ij}$, $x_{ij}$ and $y_{ij}$ are the distances to the center of $C_{ij}$ from the sensor position, and $h_{s}$ is the height of the sensor. The min–max normalization operator ${\langle \hat{x}\rangle }_{min-max}$ is defined as

$${\hat{x}}_{norm}=\frac{\hat{x}-{\hat{x}}_{min}}{{\hat{x}}_{max}-{\hat{x}}_{min}}.$$

Hence, the total representation of data from sources will have two components: the labels and their importance, which is denoted by the weights. Other source-specific weighting functions using homography of image transformations and LiDAR data acquisition models can also be used for this purpose. However, data sources like maps use uniform weights for all the cells in their FGs due to the fact that they are not measured but just extracted.

### 3.4. Integrity Analysis

The treatment of different sensors as multimodal data sources with a common frame of representation and the same dimensionality allows us to use the definitions of integrity presented in the domain of data sciences. Integrity measures overall accuracy and consistency of data sources [24]. While accuracy is defined as the correctness of validated data, consistency refers to the measure of coherence between them. Data sources with high consistency can be treated as reliable, and their integrity can be expressed as a function of coherence with respect to other data sources.

Let $S=\{s_{1},s_{2},s_{3},\cdots ,s_{N}\}$ be the set of N sensors and $s_{i}FG$ be the feature grid provided by each sensor. One cell $c_{k}$ with feature label $LB_{x}$ from $s_{i}FG$ is defined as consistent if there is at least one matching cell with $LB_{x}$ in a 3 × 3 neighborhood around the cell $c_{k}$ in $s_{j}FG$. By extension, a matching operation $f_{m}$ between FGs is defined as

(2) $$f_{m}\left(s_{i}FG,s_{j}FG\right)=N_{m}^{s_{i}FG}/N_{T}^{s_{i}FG},$$

where $N_{m}^{s_{i}FG}$ is the number of matching cells in s_*i*_FG and $N_{T}^{s_{i}FG}$ is the total number of applicable cells in $s_{i}FG$, i.e., cells with labels except LB_u_. After computing the matches between all of the possible combinations, the integrity associated with a source is computed as

(3) $$W_{i}=\frac{\sum_{\forall j,\;i\neq j}f_{m}\left(s_{i}FG,s_{j}FG\right)}{\sum_{\forall i,j\;i\neq j}f_{m}\left(s_{i}FG,s_{j}FG\right)}.$$

### 3.5. Localization Optimization

The integrity analysis mentioned in Section 3.4 assumes that the localization of a vehicle is accurately known, i.e., the localization information used in map extraction is reliable. But in real-world applications, GPS positioning—even from an inertial/dead reckoning coupled GPS receiver—can have errors due to multipath effects, outages, or drifts. Inherently, error in localization affects the consistency of map data to the other sources, hence impacting the integrity of the whole system. Hence, we developed a localization optimization procedure that uses semantic-level information from data representations of sources. It can efficiently allow integrity assessment and also identify particular defaults such as map offsets or inconsistent map sections.

In this work, a particle filter [25] is developed for map-matching to improve localization. The block diagram for the localization optimization in the ego frame of the vehicle with decision criteria is given in Algorithm 1. In the first step, new position and orientation measurements from GPS and IMU are compared with the current best localization estimate. If the new measurements ($x_{m}:\left[x_{m},y_{m},\theta_{m}\right]$) are not within the non-holonomic constraints of the current state ($X_{state}:\left[x_{state},y_{state},\theta_{state}\right]$) of the vehicle, they are detected as an outlier [26]. Conversely, consistent position and orientation measurements are used to render a map from the database and the coherence between FGs of the map and other sources is computed. If sufficient coherence is observed (greater matching than the empirically-derived threshold for $f_{m}\left(s_{i}FG,s_{j}FG\right)$ considering different sensors and scenarios), localization optimization is not performed and the data representations from each source are used for integrity assessment. In case of poor coherence between the combinations, a sequential localization optimization using particle filters is performed. The transformation function *t* on map FG (MFG) used to maximize the coherence between sources is defined as

(4) $$t\left(MFG,x,y,\theta \right)=R\left(\theta \right)*s_{i}FG+T\left(x,y\right),$$

where R(θ) is the 2D rotation matrix constructed using θ and *T* is the 2D translation vector constructed using *x* and *y* translations.

In the sequential localization optimization, coherence between the map (MFG) and each of the other sources ($s_{i}FG$) is maximized in ego frame along the *y* direction (lateral) at first by iteratively distributing particles around the best match localizations. The lateral offset estimation $y^{*}$ and the final distribution of particles from this step is used for initializing the second particle filter, which maximizes the match along the *x* (longitudinal) and θ (heading) dimensions. The resulting optimized localization ${\left(x^{*},y^{*},\theta^{*}\right)}_{s_{i}FG}$ for each $s_{i}FG$ is checked for consistency by thresholding the distance between them. If they are not consistent, the coherence between all $s_{i}FG$ is computed. An issue with the map structure is identified if the coherence between other sources (other $s_{i}FG$ combinations, e.g., LiDAR–vision) is good but ] the localization optimization of these sources cannot produce consistent localizations (within 2σ uncertainty bounds). If the localization estimations for each sensor combination are consistent, the estimation that gives the best coherence is chosen and integrity assessment is carried out. This estimation is also used to update the current localization estimation for the next time step.

**Algorithm 1** Algorithm for localization optimization.Inputs: Localization: $X_{state}$, GPS+IMU localization measurement: $x_{m}$, FG of LiDAR: *L*, FG of Vision: *C*, FG of Map: *M*, Minimum coherence limit: limit
**if**$X_{state}$ and $x_{m}$ are consistent **then** **if**
$f_{m}\left(L,M\right)>limit$ and $f_{m}\left(C,M\right)>limit$
**then**  Output: Integrity markers  Update $X_{state}$ **else**  Compute:
  
$y_{L}^{*}=\underset{y}{arg\;max}\left(f_{m}\left(L,t\left(M,0,y,0\right)\right)\right)$

  
$y_{C}^{*}=\underset{y}{arg\;max}\left(f_{m}(C,t\left(M,0,y,0\right)\right))$

  
$\left(x_{L}^{*},\theta_{L}^{*}\right)=\underset{x,\theta }{arg\;max}\left(f_{m}\left(L,t\left(M,x,y_{L}^{*},\theta \right)\right)\right)$

  
$\left(x_{C}^{*},\theta_{C}^{*}\right)=\underset{x,\theta }{arg\;max}\left(f_{m}\left(C,t\left(M,x,y_{C}^{*},\theta \right)\right)\right)$
  **if** 
${\left(x,y,\theta \right)}_{L}^{*}$ and ${\left(x,y,\theta \right)}_{C}^{*}$ are consistent **then**   Output Integrity values     Update $X_{state}$  **else**   **if** 
$f_{m}\left(L,C\right)>limit$
**then**    Output: Integrity markers    **else**    Output: Error in map     **end if**  **end if** **end if**
**else**
 Output: Error in GPS  
**end if**


## 4. Experiments and Discussions

Experiments are conducted with scenarios available in the KITTI benchmark suite [27] to establish proof of concept. Real-Time Kinematic (RTK) GPS fixes in these datasets are added with noise generated using the GPS-noise simulation model proposed by [28] to simulate poor GPS localization fixes. Outliers that are higher than the 2σ variance of the GPS-noise simulation model are used to replace RTK GPS fixes at random sections of the trajectory. Finally, 5% of the RTK GPS fixes are randomly removed from the trajectory to emulate GPS outages as they may occur in generic GPS receivers. Since different data sources have different spatial ranges, a 3D region of interest (ROI) in the vehicle’s ego frame is established. its limits in XY plane are chosen as 25 m in front of the vehicle (positive X axis), 15 meters behind (negative X axis), and 15 m at each side (Y axis). Since vision cannot provide data in the back of the vehicle as well as to the front bumper of the vehicle, the ROI of vision is limited from 3.5 m to 25 m along the positive X axis.

Even though the vision data used in this work does not cover the back view of the vehicle, the other two major sources—LiDAR and map—can provide information in the back of vehicle, hence justifying the choice of the limit in negative X axis.

The discussion on the results has three parts. Firstly, comparing the performance of the proposed method to the method in [18]. This includes a comparison of integrity markers in the datasets presented in [18] and showcasing the improvements provided by the new method in handling fault and feasibility predictors (FPs) produced by the previous method. FPs are the markers generated when the fitting of the common model to the data sources is not possible or feasible. These markers suggest the limitations of the method in [18], which mainly arise when the method is applied on non-highway scenarios. The set of five FP markers defined is

- $FP_{m}$: Not enough nodes in the map for model fitting;
- $FP_{v}$: Not enough lane markings for model fitting;
- $FP_{g}$: GPS measurement is not available or an outlier;
- $FP_{s}$: Vehicle not moving or moving very slowly;
- $FP_{t}$: Vehicle performing a hard turn.

The second part of this discussion considers more datasets in semi-urban and urban scenarios to evaluate the integrity estimation of sources in complex situations such as junctions, road splits, and merges, etc. In the final part, we compute classical integrity markers from our framework and compare them with values presented in [3].

### 4.1. Integrity Marker Comparison

**Figure 4:**
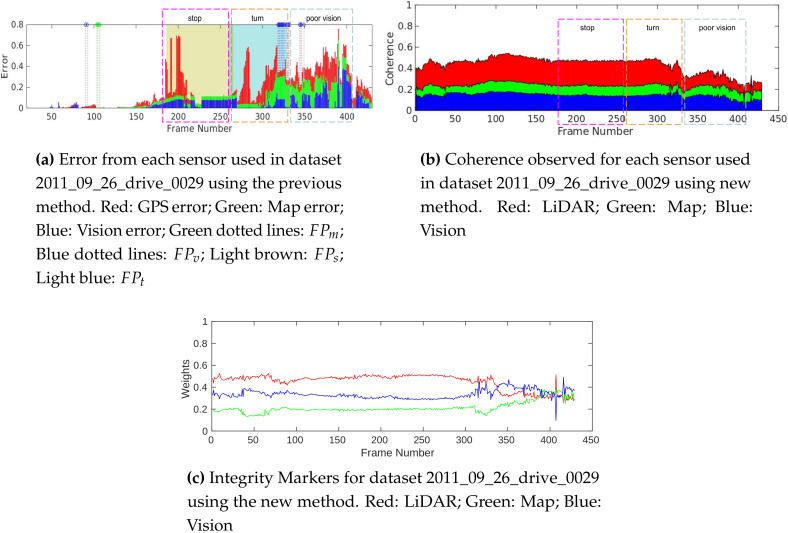
Comparison results of dataset 2011_09_26_drive _0029.

In this section, we compare the results presented in [18] with the results obtained from the new method. The key difference between these two methods is the parameter they use for the integrity computation. The former uses the error observed in model fitting to evaluate integrity, whereas the latter uses coherence between data representations to achieve the same. Hence, the contribution of error by each sensor and the contribution of coherence by each sensor are used for this analysis of the results of these methods, respectively. The same errors are introduced in the GPS for each algorithm, and the results obtained from the dataset 2011_09_26_drive_0029 are shown in Figure 4.

**Figure 5:**
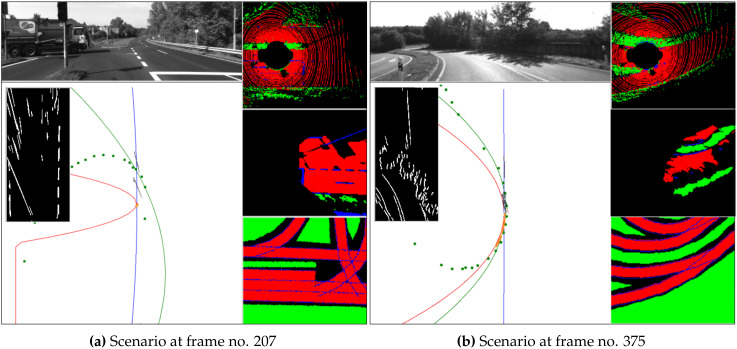
Specific scenarios from dataset 2011_09_26_drive_ 0029. Top left: view of the scenario; bottom left: model fitting; left inset: lane-marking detections; top-right: feature grid (FG) of LiDAR; middle right: FG of vision; bottom right: FG of map.

The primary advantage of the proposed method is the ability to evaluate the integrity in conditions where FPs are produced due to the limitations of the model-based integrity analysis employed in [18]. The stopping of the vehicle between frame numbers 187 and 265 and a hard left turn at the junction from 265 to 330 cause poor model extraction using the previous method, resulting in an unusable integrity evaluation. Consistent coherence is observed during the same scenario as shown in Figure 4b using the new method, providing meaningful integrity estimation. Figure 5a shows an example frame (207) in this section, where polynomial model estimation fails to represent data from sources. On the other hand, the FGs are able to represent the scenario well. After frame 330, the vehicle enters a curved link road with challenging light conditions such as shadows and oversaturated road sections, as shown in Figure 5b, causing large model-fitting errors in vision, shown in Figure 4a. Though a decrease in the coherence is observed, the addition of LiDAR and introduction of new features helps the new method provide more consistent integrity markers.

**Figure 6:**
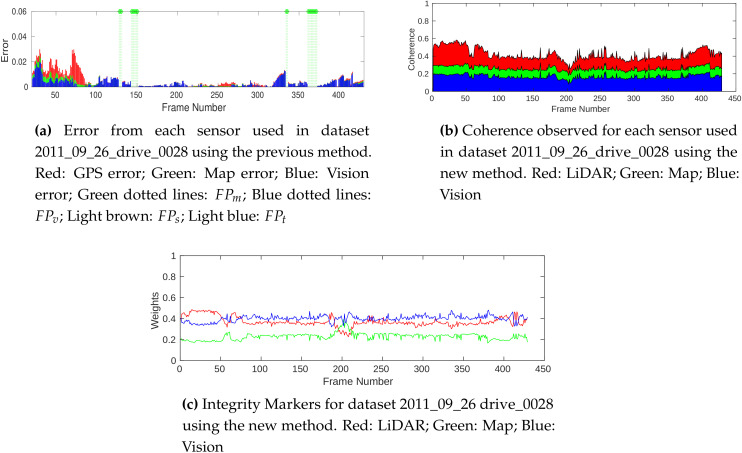
Comparison results of dataset 2011_09_26_drive _0028.

In Figure 6, the results of integrity assessment in a highway scenario are presented, where the old method reliably performed. The $FP_{m}$ instances observed in this dataset are due to the lack of map nodes to reliably fit the polynomial model in straight line road sections. In the new method, the model fitting is replaced with FG data representation, which eliminates such errors in modeling.

The comparison of integrity markers in specific cases presented in [18] with the integrity markers provided by the new method is given in Table 1. A general tendency of improved integrity values is observed across all datasets and scenarios. For example, in the second row of Table 1, the integrity weight of vision computed using the old method was lower due to the improper detection of curved lane markings as straight lane markings. This resulted in an inconsistent polynomial model compared to the other two data sources, causing a low integrity weight of 0.175. But using the new method, drivable road detection along with surrounding structure detection improved the consistency of vision data with other sources, resulting in a higher integrity value of 0.612. The proposed method is proven to be able to handle every situation where FP was provided by the old method. In the first row of Table 1, the lack of sufficient map nodes on a straight road segment made model-based integrity estimation impossible, as confirmed by the $FP_{m}$ flag. The new approach enables integrity estimation and provides an integrity weight of 0.422. It is worth noting that a high integrity value is not observed because of poor map rendering due to lack of correct lane width information from the map. Incorrect road segment selection from the map does not affect the new method as it uses all of the neighborhood roads in integrity estimation.

### 4.2. Complex Situations

**Figure 7:**
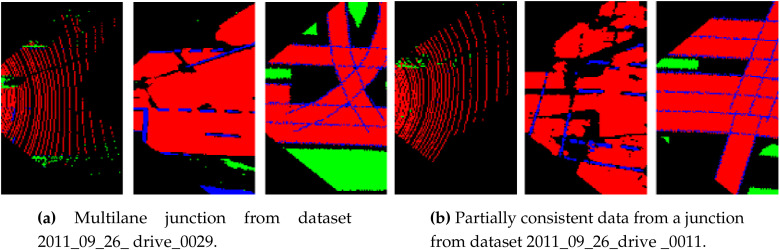
Examples of complex scenarios—cells with road labels(red), lane marking labels (blue), other surface labels (green), unclassified labels (black).

This section is dedicated to analyzing the behavior of the integrity assessment system in some of the selected complex scenarios present in the KITTI dataset. In Figure 7a, an example of a semi-urban road junction is shown. Due to the lack of information from the map, the rendering process failed to reconstruct the continuity of lanes at the intersections. On the other hand, vision and LiDAR data detected all of the branch roads at the junction and managed to perceive the width of each of these road sections accurately. This results in a lower integrity value for the map at this junction (Frame numbers: 310–320) compared to other sources, as shown in Figure 4c.

One of the main reasons behind the proposed data representation is the fact that it is an improvement over other existing geometrical models for intersections, which fail to accommodate partially correct data. Figure 7b shows a partial road detection from LiDAR due to the difference in elevation of one of the road branches in the scenario. Even though data available from LiDAR is not complete, the part that is detected is coherent with both vision and map. In fact, LiDAR has more integrity than vision in the comparison, not only because of its coherence in road detections, but also, the available grass-patch detection compensates the partial road detection. The integrity values in this scenario (Frame numbers: 120–200, dataset 2011_09_26_drive_0011) are computed around 0.456, 0.349, and 0.165 for LiDAR, vision, and map, respectively.

### 4.3. Performance of Integrity Monitoring

**Figure 8:**
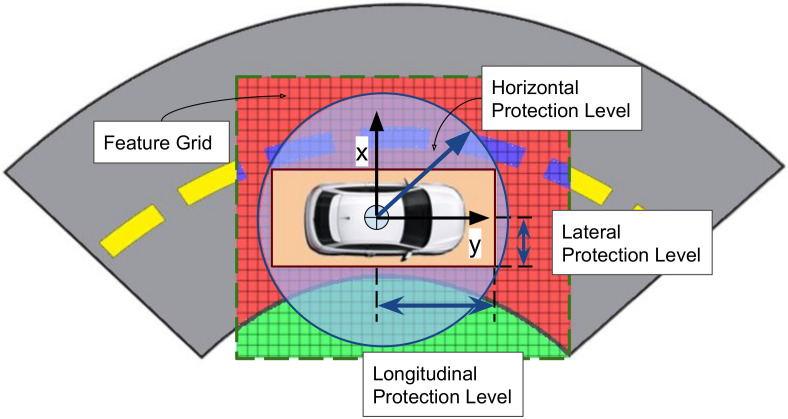
Illustration of protection levels for the localization of ground vehicles.

To evaluate and compare the proposed integrity framework to the integrity concepts transposed from civil aviation concepts, the Horizontal Protection Level (HPL) is computed. According to [29], the HPL is the radius of a circle in the horizontal plane that describes the region assured to contain the indicated horizontal position. It is the statistical bound for horizontal position error with a confidence level derived from the integrity risk requirement of an application. We also compute the Lateral Protection Level (LatPL) and Longitudinal Protection Level (LonPL), as proposed in [3]. The illustration given in Figure 8 shows the geometrical interpretations of these protection levels with respect to the ego frame of the vehicle and feature grids. Extending these concepts, we use the final distribution of the particles from the localization optimization particle filter described in Section 3.5 to compute LatPL, LonPL, and HPL. The lateral and longitudinal positions of all the particles that belong to the 95th percentile of the coherence matching scores are modeled using a Gaussian distribution. LatPL, LonPL, and HPL are then computed using the average standard deviation of particle distributions from each sensor combination used to optimize localization as

(5) $$LatPL=2\sqrt{\left(\sigma_{CY}^{2}+\sigma_{LY}^{2}\right)/2},$$

(6) $$LonPL=2\sqrt{\left(\sigma_{CX}^{2}+\sigma_{LX}^{2}\right)/2},$$

(7) $$HPL=2\sqrt{\left(\sigma_{CX}^{2}+\sigma_{CY}^{2}+\sigma_{LX}^{2}+\sigma_{LY}^{2}\right)/4},$$

where $\sigma_{CX}^{2}$ and $\sigma_{CY}^{2}$ are the lateral and longitudinal variances of particles from the vision-map optimization result and $\sigma_{LX}^{2}$ and $\sigma_{LY}^{2}$ are the lateral and longitudinal variances of particles from the LiDAR-map optimization result.

**Figure 9:**
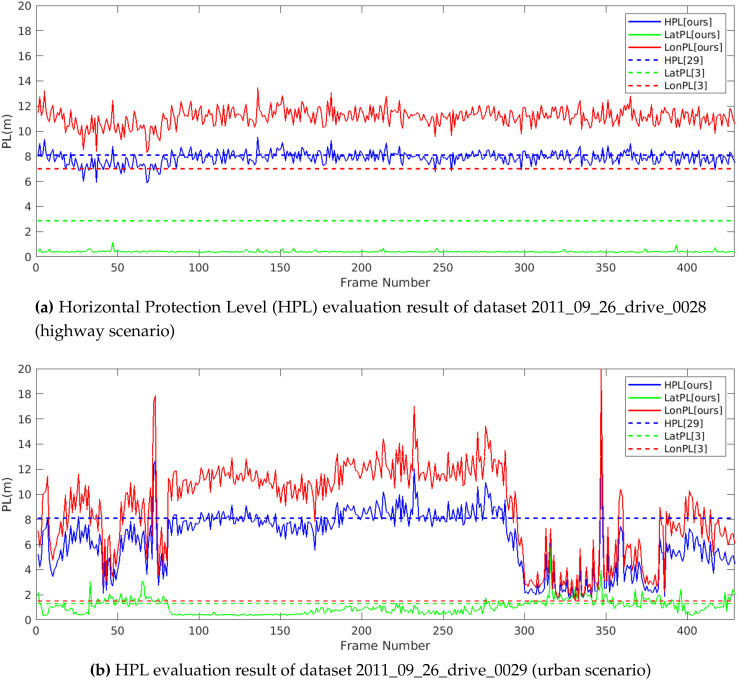
Horizontal Protection Level (HPL) comparison.

The results obtained from the HPL evaluation of two of the datasets presented in Section 4.1 are shown in Figure 9. Using historical HPL data available from hte European Global Navigation Satellite System Agency [30], the average value of the HPL over the last 5 years (from 01-2015 to 07-2020) for the nearest zone (Zurich) to the dataset location (Karslruhe) is calculated as 8.1 m. According to the total integrity levels and allocation of integrity risks derived in [3], [LatPL,LonPL] of the perception block is computed as [2.85 m, 7 m] for highway scenarios and [1.45 m, 1.45 m] for non-highway scenarios. The results of this comparison are shown in Figure 9. In highway scenarios, the LatPL computed using our method is completely within the LatPL limit derived by [3], whereas in urban scenarios, 91% of the time, LatPL from our method is under the limit. On the other hand, the HPL computed using our method shows good coherence with the historical HPL calculated using [30]. However, the LonPL computations are, most of the time and in both scenarios, outside the limit of LonPL derived by [3]. This is due to the fact that the sensors considered in this work are better at providing lateral information ([3]) than longitudinal information. This is evident from the highway scenario in Figure 9a, where the road is straight without any other significant information to bound the sensor data in the longitudinal direction. In Figure 9b, sections where the LonPL computed from our method is closer to the LonPL limit of 1.45 m contain curved road sections or other distinguishable surfaces, which helps to reduce LonPL considerably. Hence, the results presented in this section demonstrate the capability of the proposed method to assess the integrity of perception sensors in localizing vehicles with the accuracy required for urban and highway navigation.

## 5. Conclusions

This work presents a framework for integrity monitoring of sources used in the localization of autonomous vehicles. The limitations of common geometrical models in representing multimodal data sources are identified in this work. To overcome these issues, a semantic feature grid model is proposed that can geometrically represent different features using labels. A function for coherence evaluation between feature grids is formalized to iteratively optimize the localization as well as to assess the integrity of data sources. The framework is tested using different scenarios from datasets, and the results show the versatility of the proposed model, which is able to provide reliable and consistent integrity estimation in highway as well as semi-urban and urban environments. This method is proven robust against inconsistencies in feature detections such as partial detections, occlusions, and poor map rendering. The method presented claims scalability since it can be implemented with any number of sensors and digital map sources. The only requirement for the applicability of this framework is the ability to detect common features from all of the data sources and represent them geometrically in the proposed feature grid representations. This work also illustrates how classical integrity markers like protection levels can be transposed for perception data sources used in autonomous vehicles.

## 6. Future Works

The rule-based map rendering technique used in this method is observed to be contributing several inconsistencies, which makes it difficult to isolate map rendering errors from GPS positioning errors. We propose the use of high-definition maps, which are enriched with globally localized lane-level information, to address this issue. Accurate maps will improve the coherence estimation between features detected from other data sources such as stop lines, pedestrian crossings, lane merging information, road structure information, etc. It will also be important to study map-rendering techniques that improve integrity multi-source perception analysis by including precise building footprints, road width information, lane markings, and traffic sign localization.

## Figures and Tables

**Table 1:** Results obtained using the proposed method and the method presented in [18].

Dataset–Frames	Integrity [18]	Integrity (Ours)	Situation
Dataset 1–150	$FP_{m}$	map-0.422	not enough nodes from the map
Dataset 1–21	vision-0.175	vision-0.612	no good quality lane markings
Dataset 2–390	map-0.087	map-0.374	road with multiple curvatures
Dataset 3–562	$FP_{v}$	vision-0.573	partial occulusion in vision due to vehicles
Dataset 3–1117	map-0.006	map-0.381	wrong map extraction
Dataset 4-22	vision-0.214	vision-0.681	road with multiple curvatures
Dataset 4-260	vision-0.651	vision-0.629	highway road with single curvature
